# Inhibition of *Candida albicans* Biofilm Formation by the Synthetic Lactoferricin Derived Peptide hLF1-11

**DOI:** 10.1371/journal.pone.0167470

**Published:** 2016-11-30

**Authors:** Paola Morici, Roberta Fais, Cosmeri Rizzato, Arianna Tavanti, Antonella Lupetti

**Affiliations:** 1 Department of Translational Research and New Technologies in Medicine and Surgery, University of Pisa, Pisa, Italy; 2 Department of Biology, University of Pisa, Pisa, Italy; Laurentian, CANADA

## Abstract

The aim of this study was to evaluate the *in vitro* activity of the synthetic peptide hLF1-11 against biofilm produced by clinical isolates of *Candida albicans* with different fluconazole susceptibility. The antibiofilm activity of the peptide hLF1-11 was assessed in terms of reduction of biofilm cellular density, metabolic activity and sessile cell viability. The extent of morphogenesis in hLF1-11 treated and untreated biofilms was also investigated microscopically. Transcription levels of genes related to cell adhesion, hyphal development and extracellular matrix production were analysed by qRT-PCR in hLF1-11 treated and untreated biofilms. Exogenous dibutyryl-cAMP (db-cAMP) was used to rescue morphogenesis in cells exposed to the peptide. The results revealed that hLF1-11 exhibited an inhibitory effect on biofilm formation by all *C*. *albicans* isolates tested in a dose-dependent manner, regardless of their fluconazole susceptibility. Visual inspection of treated or untreated biofilm cells with an inverted microscope revealed a significant reduction in hyphal formation by hLF1-11 treated cells, as early as 3 hours of incubation. Moreover, hLF1-11 showed a reduced activity on preadherent cells. hLF1-11 induced the down-regulation of biofilm and hyphal-associated genes, which were predominantly regulated via the Ras1-cAMP-Efg1 pathway. Indeed, exogenous db-cAMP restored morphogenesis in hLF1-11 treated cells. The hLF1-11 peptide significantly inhibited biofilm formation by *C*. *albicans* mainly at early stages, interfering with biofilm cellular density and metabolic activity, and affected morphogenesis through the Ras1-cAMP-Efg1 pathway. Our findings provide the first evidence that hLF1-11 could represent a potential candidate for the prevention of biofilm formation by *C*. *albicans*.

## Introduction

*Candida albicans* is an opportunistic yeast, responsible for systemic infections in individuals with impaired immune response [1]. Nosocomial *C*. *albicans* infections are often related to the ability to produce biofilm on mucosal surfaces and implanted medical devices [2–4]. Biofilm formation is a finely regulated process, which involves multiple interconnected signalling pathways [5], leading to a structured microbial community that is attached to a surface and embedded in an exopolymeric extracellular matrix [2,6,7]. The polysaccharide matrix acts as a barrier for sessile cells, preventing the entrance of most commonly used antifungal agents, thus conferring drug resistance. Indeed, sessile *C*. *albicans* cells are up to 1000-fold more resistant to common antifungals than planktonic cells, even in the absence of specific drug-resistance genes [8,9]. Thereby, biofilm is a reservoir of viable fungal cells potentially leading to systemic infections, with a mortality rate of 40–60% [10,11].

Increasing efforts are currently underway in developing alternative strategies aimed at eradicating biofilm-related infections. Among these, antimicrobial peptides (AMPs) have been widely investigated as novel therapeutic agents [12,13].

Human lactoferrin (hLF) is an iron-binding glycoprotein (77 kDa) synthesized by mucosal gland epithelial cells and neutrophils in response to inflammatory stimuli [14]. By pepsinolysis, hLF releases lactoferricin H (residues 1 to 47) from its N-terminus, which contains two cationic domains (residues 2–5 and 28–31) [15,16]. A synthetic peptide comprising the first cationic domain of lactoferricin H, further referred to as hLF1-11, possesses a high antibacterial and antifungal activity, with a more pronounced effect in vivo [17,18]. Furthermore, hLF1-11 exerts modulatory effects on immune cells stimulating macrophages and dendritic cells, thus contributing to the clearance of infections [19,20]. In addition, previous studies have shown that hLF1-11 has no cytotoxic effects against human erythrocytes, and its administration to healthy volunteers was well tolerated [21].

Until now, the exact mechanisms of action of hLF1-11 are not fully characterised, despite mitochondria seem to be a target of hLF1-11 in *C*. *albicans* [22].

To date, no evidence is available on the role played by the hLF1-11 peptide on biofilm production by *C*. *albicans*, although immobilizing hLF1-11 onto titanium surfaces of implants reduced bacterial adhesion and biofilm formation [23].

This study was aimed at i) evaluating the potential inhibitory activity of hLF1-11 against biofilm formation of clinical isolates of *C*. *albicans* characterised by different fluconazole susceptibility, and ii) improving our understanding of the mechanism(s) underlying the hLF1-11-induced antibiofilm activity.

## Materials and Methods

### Strains

A panel of *Candida albicans* strains including ten clinical isolates collected at the U. O. Microbiologia Universitaria, Azienda Ospedaliero-Universitaria Pisana (Pisa, Italy) and the reference strain SC5314 were investigated for biofilm production. Furthermore, fluconazole susceptibility was determined by Vitek 2 system (AST-YS07 card; bioMérieux, Marcy l’Étoile, France). All strains were stored in YPD broth (Yeast Peptone Dextrose, Difco BD, Milan, Italy) supplemented with 40% (vol/vol) glycerol at -20°C and -80°C, subcultured at 37°C on YPD agar plates, and kept at 4°C until testing. After 16h incubation at 30°C, cells were washed in sodium phosphate buffer (NaPB, pH 7) and suspended at the desired concentration.

### Lactoferrin peptide

The synthetic peptide corresponding to the first eleven residues of human lactoferrin (GRRRRSVQWCA; molecular mass, 1374.6 Da), further referred to as hLF1-11, was purified by Peptisyntha Inc (Torrance, CA, USA), with purity exceeding 95%. Stocks (10mM in 0.01% acetic acid; pH 3.7) were stored at -20°C.

### hLF1-11-induced candidacidal activity on planktonic cells

The hLF1-11 antifungal activity was tested by the broth microdilution method according to CLSI standard M27-A3 with minor modifications [24]. Briefly, a final suspension of 1×10^3^ cells/ml in RPMI 1640 medium (diluted 1:4 in NaPB) was adjusted to pH 7 with 0.165 3-(N-morpholino)-propanesulfonic acid (MOPS; Sigma Aldrich, St. Louis, USA) and inoculated in polystyrene, round-bottomed, 96-well microtiter plates (Falcon, Becton Dickinson BD, Milan, Italy) to reach a final volume of 100μl/well. hLF1-11 concentrations tested were 0.17-176mg/L. Following incubation at 37°C for 24h, MIC values were determined as the lowest peptide concentration inhibiting fungal growth. Two sets of independent experiments were performed, each in duplicate.

### Inhibition of biofilm formation

Fungal suspensions were prepared at 2×10^6^ cells/ml in four-fold diluted RPMI 1640 medium supplemented with glucose (2% final concentration) and buffered with MOPS. An aliquot of each yeast suspension was transferred in polystyrene, flat-bottomed, 96-well microtiter plates and incubated with different hLF1-11 concentrations (44, 88, 176mg/L) at 37°C for 24h, in a final volume of 100μl/well. The inhibitory activity of hLF1-11 on *C*. *albicans* biofilm formation was also evaluated at earlier time points, including 1.5h, 3h, 6h and, as control, 24h, using a peptide concentration of 88mg/L. A positive and negative control were included in each experiment.

After incubation, non-adhered cells were removed by washing twice with phosphate buffered saline (PBS). The antibiofilm activity of hLF1-11 was evaluated by i) spectrophotometrically measuring the biofilm cellular density; (ii) quantifying metabolic activity by XTT assay, and (iii) counting viable sessile cells (CFU/ml) after biofilm scraping from wells. Three independent experiments were performed, each in triplicate.

### Biofilm cellular density

Biofilm cellular density formed in the presence or absence of the peptide was determined by measuring the optical density at 490 nm (OD_*λ*490nm_) using an automated plate reader (Model 550 Microplate Reader Bio-Rad, Milan Italy). Background optical density was subtracted from the values measured in each well.

### XTT assay

XTT solution was prepared at 0.5g/L in PBS buffer and mixed with a menadione solution dissolved in acetone at a final concentration of 1μM. An aliquot of 100μl of this solution was inoculated into each well containing dry preformed biofilms and the 96-well plate was incubated in the dark at 37°C for 2h. Next, the supernatant (80μL) was transferred into a 96-well plate to measure colorimetric changes at 490 nm (Model 550 Microplate Reader).

### Sessile cell viability

The recovery of sessile cells from biofilm formed on the well bottom was next evaluated. Briefly, after washings, 200μl of PBS was added to the wells and adhered cells were detached by scraping with the micropipette tip. Recovered cells were transferred to tubes containing 800μl of PBS and vortexed for 5 minutes. Suspensions were then sonicated (VWR Ultrasonic Cleanear, 230V/50-60Hz), vortexed, and serially diluted. Aliquots (200μl) of each dilution were plated on YPD agar and colony counts were performed after 24h incubation at 37°C.

### hLF1-11 activity on *C*. *albicans* cell morphology

In order to assess the hLF1-11-induced effect on the architecture of biofilm formed by *C*. *albicans*, biofilms produced by four representative *C*. *albicans* strains (SC5314, CA22, CA37 and CA688) were visualised under an inverted microscope (Olympus IMT-2) at 400× magnification, following 24h exposure to the peptide.

### Activity of hLF1-11 on pre-adhered *C*. *albicans* cells

*C*. *albicans* SC5314 (1×10^6^ cells/mL) was suspended in diluted RPMI 1640 supplemented with 2% glucose and buffered with MOPS. Aliquots (100μL) of fungal suspension were inoculated into 96-well plates, and incubated for 1.5h or 6h permitting fungal adhesion. In parallel, plates were incubated for 24h to allow complete biofilm development, acting as a positive control. Following incubation, non-adhered cells were removed by washing with sterile PBS, and hLF1-11 (100μL) at various concentrations (44, 88 and 176mg/L) was added to each well. Next, plates were further incubated for 24h at 37°C for biofilm formation. Subsequently, evaluation of biofilm cellular density, metabolic activity, and cell viability was carried out as previously described. Three independent experiments were performed, each in triplicate.

### Quantitative real time RT-PCR (qRT-PCR) analysis of *C*. *albicans* biofilm-related genes

qRT-PCR was used to investigate hLF1-11-induced changes in transcription level of genes related to biofilm formation. Genes involved in the Ras1-cAMP-Efg1 pathway (*RAS1*, *EFG1*, *CYR1*), MAP kinases pathway (*HST7*), Cph2-Tec1 pathway (*TEC1*), hyphal-specific genes (*ALS3*, *HWP1*, *ECE1*), and genes related to the production of extracellular matrix (*GSC1*, *ZAP1*, *ADH5* and *CSH1*) were evaluated. *C*. *albicans* SC5314 cells (1×10^6^ cells/mL) were grown in the absence or presence of hLF1-11 (88 mg/L) in 24-well plates for 24 h at 37°C. Next, wells were washed with PBS and adhered cells were removed from the bottom of the wells by scraping (5 wells/sample). Total RNA was extracted from sessile cells with the Nucleospin RNA (Macherey Nagel, Duren, Germany) according to manufacturer’s instructions and stored at -80°C. The quality and quantity of the extracted RNA were determined spectrophotometrically. Total RNA (1μg) was converted into cDNA with random primers in a 20μL reaction volume, using the Reverse Transcription System kit (Promega), following manufacturer’s instructions. Primer sequences used for amplification of specific genes are shown in S1 Table. qRT-PCR mixtures contained 6μL cDNA, 10μL SYBR Green Master Mix (Applied Biosystem, Life technologies, Monza, Italy), 1pMol/μL of each primer, and sterile MilliQ water to a final volume of 20μL. qRT-PCR was performed in a 96-well plates on CFX96 Touch Real-Time PCR Detection System (BioRad) (95°C for 60s, followed by 40 cycles of 95°C for 5s, 60°C for 30s). Actin (*ACT1*) was used as internal control. The transcription level of the selected genes was calculated using the formula of 2^-ΔΔCt^. Three independent experiments were performed, each in triplicate.

### cAMP rescue experiments

In order to verify the role of hLF1-11 in the inhibition of Ras1-cAMP-Efg1 pathway, the effect of exogenous dibutyrylcAMP (db-cAMP; Sigma Aldrich, Milan Italy) on fungal cells treated with the peptide was evaluated. Briefly, overnight *C*. *albicans* SC5314 cells (1×10^6^ cells/mL) were aliquoted into wells of a 24-well plate (1 mL/per well). A100 mM stock of db-cAMP solution was prepared in sterile water and stored at -20°C. Db-cAMP was added to the fungal suspension to a final concentration of 5mM, immediately following the addition of 44mg/L hLF1-11. The untreated cells with or without db-cAMP served as control. Each sample was tested in triplicate. After incubation at 37°C for 4h, cells were recovered (5,000×g for 5min) and visualised (Olympus) at a 400× magnification. The percentage of cells growing as yeast, pseudohyphae and hyphae was determined from a mean of three replicate wells.

### Statistical analysis

Data were expressed as means±standard error of the mean (S.E.M.). Results were evaluated by one-way ANOVA test, followed by the Tukey-Kramer post-hoc test, using GraphPad Instat software (version 6.05 for Windows, La Jolla, CA USA). The level of significance was set at a *P* value of < 0.05.

## Results

### hLF1-11-induced candidacidal activity on planktonic cells

The hLF1-11-induced candidacidal activity on planktonic cells of ten clinical isolates and the reference strain SC5314 was evaluated (Table 1). All strains exhibited similar susceptibility to hLF1-11 with MIC values ranging from 22 to 44 mg/L (Table 1). No difference in hLF1-11 MIC values was found between fluconazole-resistant (CA688), and -susceptible clinical isolates (Table 1).

**Table 1:** MICs of fluconazole and hLF1-11 against planktonic cells of *C*. *albicans* clinical isolates and the reference strain SC5314.

Strain	Source	Fluconazole MIC (mg/L)^a^	hLF1-11 MIC (mg/L)
SC5314	Reference strain	≤1 (S)	22
CA28	Blood culture	≤1 (S)	22
CA688	Bronchoalveolar lavage	64 (R)	22
CA22	Oral swab	≤1 (S)	22
CA5	Oral swab	≤1 (S)	44
CA31	Oral swab	≤1 (S)	44
CA37	Vaginal swab	≤1 (S)	44
CA3	Vaginal swab	≤1 (S)	22
CA17	Vaginal swab	≤1 (S)	22
CA18	Vaginal swab	≤1 (S)	44
CA35	Vaginal swab	≤1 (S)	22

^a^Interpretation of fluconazole susceptibility was performed according to the EUCAST (2015) clinical interpretive breakpoints: ≤2mg/L Susceptible (S), >4mg/L Resistant (R)

### hLF1-11 antibiofilm activity

**Fig 1:**
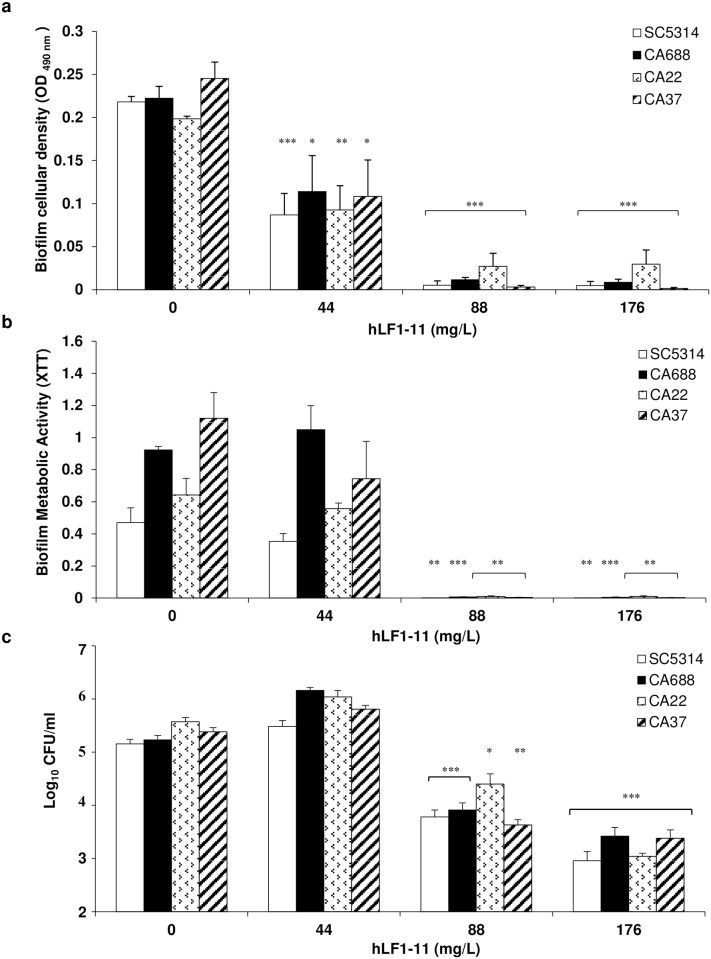
Effect of hLF1-11 on biofilm formation by four representative *C*. *albicans strains*. *C*. *albicans* cells (1x10^6^ cells/ml) were co-incubated with various concentrations of hLF1-11 for 24h at 37°C. After incubation, the antibiofilm activity of the peptide was assessed in terms of (a) biofilm cellular density reduction, (b) metabolic activity by the XTT assay, and **c** reduction of sessile cell viability. Data are expressed as the mean of three independent experiments ± SEM. SC5314 open bars, CA688 closed bars, CA22 dotted bars, CA37 diagonally hatched bars * *P*≤0.05, ** *P*<0.01, *** *P*<0.001, as compared to the untreated control biofilms.

The antibiofilm activity of hLF1-11 was evaluated against *C*. *albicans* reference strain SC5314 and on the clinical isolates (CA688, CA37, CA22) previously selected based on different RAPD profiles (data not shown), propensity to produce biofilm (data not shown), and fluconazole susceptibility (Table 1). Fungal cells were incubated in the absence and presence of different peptide concentrations (44, 88 and 176mg/L). hLF1-11 induced a significant reduction in biofilm cellular density, metabolic activity and number of viable cells, (*P<*0.05), in all the tested strains (Fig 1). The antibiofilm activity was shown to occur in a dose-dependent manner. The results revealed a 50% reduction in cellular density values after incubation with 44 mg/L hLF1-11, compared to the untreated biofilm and a 100% reduction with doses higher than 88mg/L (Fig 1a). Furthermore, a significant reduction in metabolic activity was observed in the presence of the peptide at 88 and 176mg/L (100%; *P* <0.05), compared to the untreated control (Fig 1b), while no statistically significant reduction was observed at the lowest concentration (44mg/L). The hLF1-11 peptide also induced 1- and 2-log reduction in cell viability (CFU/mL) at 88 and 176mg/L, respectively (Fig 1c). No decrease in cell viability was observed at the lowest dose of hLF1-11 tested.

### Kinetics of hLF1-11 antibiofilm activity

**Fig 2:**
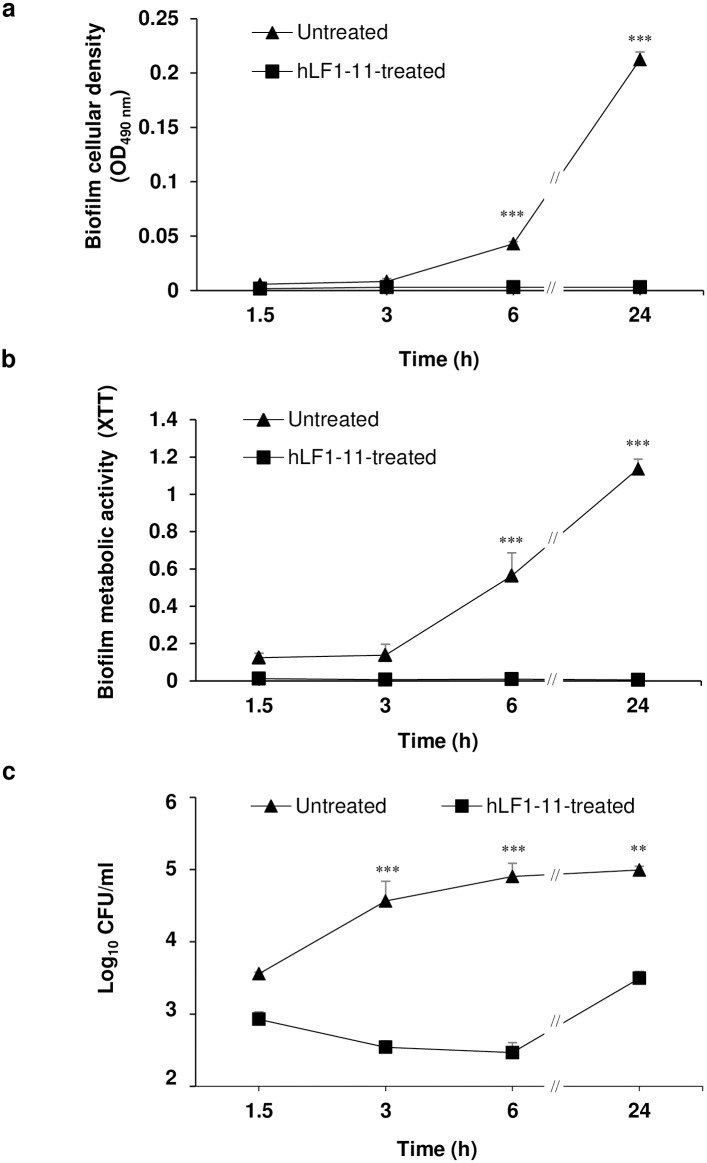
Kinetics of hLF1-11 activity on biofilm formation by four representative *C*. *albicans* strains. *C*. *albicans* SC5314 cells (1x10^6^ cells/ml) were co-incubated with hLF1-11 (88mg/L) for different time periods (1.5, 3, 6 and 24h) at 37°C. Following incubation, the antibiofilm activity of the peptide was assessed in terms of (a) biofilm cellular density reduction, (b) metabolic activity by the XTT assay, and (c) reduction of sessile cell viability. Data are expressed as the mean of three independent experiments ± SEM. hLF1-11-treated sample (square symbol), untreated sample (triangle symbol) ** *P*<0.01, *** *P*<0.001, as compared to the untreated control biofilms.

In order to assess whether the hLF1-11 antibiofilm activity after a 24-hour incubation could also be observed at earlier stages, the peptide (88mg/L) and fungal cells were incubated for 1.5h, 3h, 6h and, as a control, 24h. Evaluation of early antibiofilm activity was performed considering the same parameters as previously described. Data obtained from kinetic experiments demonstrated that hLF1-11 induced a reduction in biofilm formation after a 3h incubation (Fig 2a and 2b). Interestingly, a complete abolishment of biofilm metabolic activity was observed as early as 1.5h, which persisted up to 24h. In addition, hLF1-11 caused a 2-log reduction in sessile cell viability after a 3h incubation period, compared to untreated biofilms. This reduction decreased to 1-log following a 24h incubation period.

### hLF1-11 effect on *C*. *albicans* cell morphology

**Fig 3:**
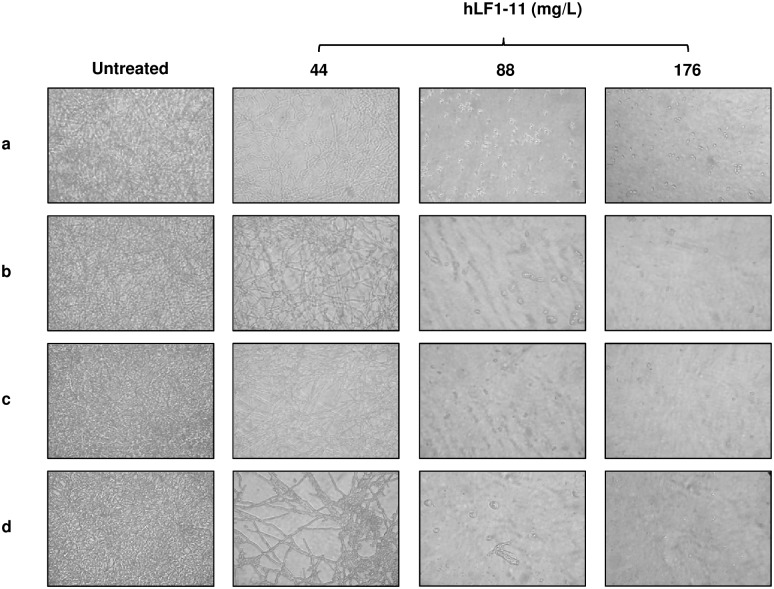
Effect of hLF1-11 on biofilm architecture of *C*. *albicans* strains. Inverted microscope images (400× magnification) of (a) *C*. *albicans* SC5314 and three clinical isolates, (b) CA688, (c) CA22, and (d) CA37, untreated and treated with 44mg/L, 88mg/L and 176mg/L hLF1-11. *C*. *albicans* strains were co-incubated with the different hLF1-11 concentrations at 37°C for 24h.

The hLF1-11 effect on biofilm architecture of the 4 *C*. *albicans* strains was evaluated using an inverted microscope, following a 24h incubation in the presence/absence of the peptide. The peptide induced a dose-dependent reduction in the number of hyphae compared to untreated cells for all the strains tested (Fig 3a–3d). Indeed, microscopic observation in the absence of the peptide revealed a very thick layer of mature biofilm, consisting of hyphae and matrix exopolysaccharide. In the presence of hLF1-11 at 44mg/L the biofilm layer appeared less compact and homogeneous, with isolated hyphae. At higher concentrations (88 and 176mg/L), the peptide completely inhibited biofilm formation, with fungal cells predominantly growing as yeast.

### hLF1-11 effect on pre-adhered *C*. *albicans* cells

**Fig 4:**
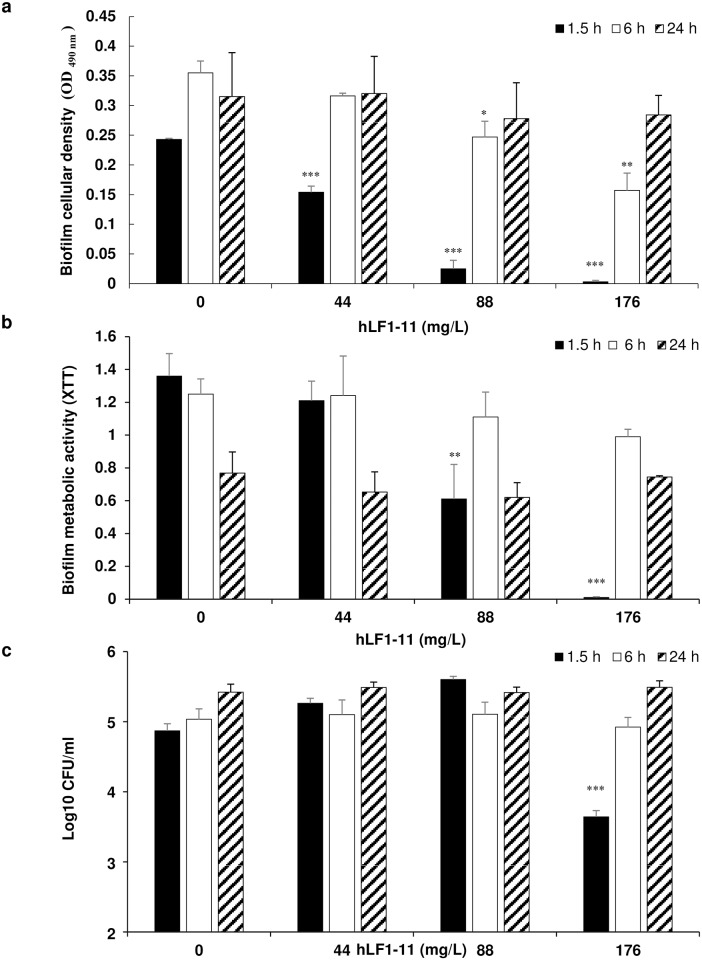
Effect of hLF1-11 on pre-adhered *C*. *albicans* cells. *C*. *albicans* SC5314 cells were allowed to adhere for different times (1.5h, 6h and 24h) and then various concentrations of hLF1-11 were added and incubated for 24h at 37°C. Following incubation, the antibiofilm activity of the peptide was assessed in terms of (a) biofilm cellular density reduction, (b) metabolic activity by the XTT assay, and (c) reduction of sessile cell viability. Data are expressed as the mean of three independent experiments ± SEM. 1.5h closed bars, 6h open bars, 24h diagonally hatched bars * *P*≤0.05, ** *P*<0.01, *** *P*<0.001, as compared to the untreated control biofilms.

In order to evaluate whether the peptide could act on pre-adhered cells, cells were incubated for 1.5 or 6h to allow adhesion to the 96-well plate or, as a control, 24h, prior to addition of different hLF1-11 concentrations (44, 88, and 176mg/L) and incubated for further 24h at 37°C. The results obtained indicate that the addition of hLF1-11 to *C*. *albicans* cells after 1.5h adhesion induced a significant dose-dependent decrease in cellular density and metabolic activity of the biofilm formed and about 1.5 log reduction (*P*<0.001) in colony count compared to the control with the highest peptide concentration tested (176mg/L). Addition of hLF1-11 to cells after a 6h adhesion phase revealed a significant decrease in biofilm cellular density at the concentrations of 88 and 176mg/L (Fig 4a), but no decrease in biofilm metabolic activity or in the number of sessile viable cells at all the concentrations tested (Fig 4b and 4c). As expected, no change was observed on preformed biofilm (Fig 4a–4c).

### Transcriptional profiles of biofilm associated genes

**Fig 5:**
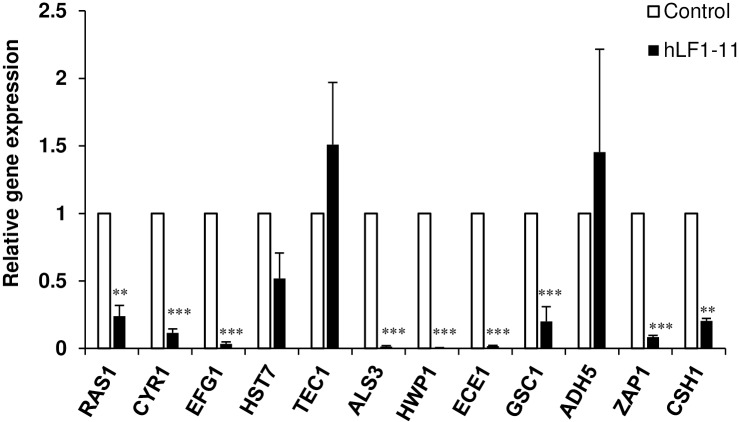
Quantitative real time RT-PCR analysis of *C*. *albicans* biofilm-related genes. *C*. *albicans* SC5314 biofilms were treated with 88mg/L of hLF1-11 in 24-well plates for 24h at 37°C and the expression of the target genes was determined qRT-PCR. Level of gene expression (closed column) is presented as fold change relative to the control group (untreated biofilms, open column). *ACT1* expression was used for normalization. Assays were performed in triplicate and bars represent means ± S.E.M from three independent experiments. ** *P*<0.01 *** *P*<0.001.

The hLF1-11-induced changes at the transcriptional level of twelve genes related to biofilm formation was assessed by qRT-PCR. The expression level of each gene was analysed in cells exposed to the peptide (88mg/L) for 24h and compared to untreated cells (Fig 5). Analysis of the actin-normalised data showed that the peptide induced a significant decrease in expression level of the following genes: *RAS1*, *CYR1*, *EFG1*, *ALS3*, *HWP1*, *ECE1*, *GSC1*, *ZAP1*, and *CSH1*, while hLF1-11 did not influence the expression of *HST7*, *TEC1*, and *ADH5* genes. Furthermore, the latter showed a variable level of expression compared to the untreated control, in all experimental replicates.

### Rescue of morphogenesis by exogenous cAMP

**Fig 6:**
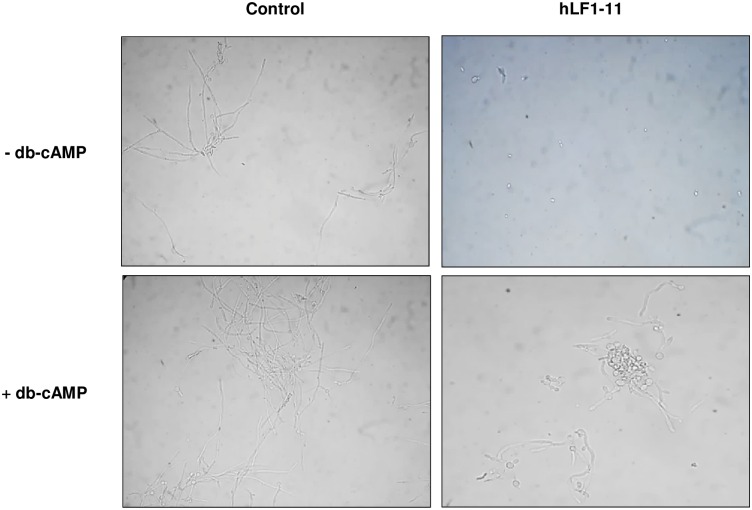
Exogenous db-cAMP restored the hLF1-11-inhibited hyphal formation. A cell suspension of *C*. *albicans* SC5314 (1x10^6^ cells/ml) was incubated at 37°C with db-cAMP (5mM) immediately following the addition of 44mg/L hLF1-11. Peptide free cells with or without db-cAMP served as control. After 4h, cells were recovered from the wells by centrifugation and visually inspected by microscopy (400 × magnification).

To verify the role of hLF1-11 in Ras1-cAMP-Efg1 pathway inhibition, exogenous db-cAMP was administered to fungal cells immediately after treatment with the hLF1-11 peptide (44mg/L) and microscopically evaluated after a 4h incubation. hLF1-11-treated cells used as a control were mainly in the yeast form (Fig 6). db-cAMP addition to hLF1-11-treated cells resulted in the recovery of filamentous growth. Fungal cell counts revealed a predominance of cells in the yeast form (80%), with a few pseudohyphae (20%) and no hyphae in the hLF1-11-treated sample, without exogenous db-cAMP. Notably, incubation with db-cAMP partially restored morphogenesis, with a percentage of filamentous forms (pseudohyphae and hyphae) rising to 60% (data not shown).

## Discussion

The main finding from the present study demonstrates that hLF1-11 can significantly inhibit *C*. *albicans* biofilm formation. This conclusion is based on the following findings. First, a reduction of total cellular density, metabolic activity and cell viability was observed in a dose-dependent manner after a 24-hour incubation. Second, kinetics experiments indicated that hLF1-11 inhibits early stages of biofilm formation in *C*. *albicans*, starting from 3h, where an approximate 2-log reduction in sessile cell viability was observed, although a significant reduction of all biofilm parameters was shown after 6h.

However, the hLF1-11 inhibitory effect was only observed in biofilm cellular density in the presence of pre-adhered cells (1.5-6h). Moreover, *C*. *albicans* mature biofilm was unaffected following a 24h incubation with the peptide. Accordingly, other antimicrobial peptides, such as β-peptides and LL37, exerted a direct antibiofilm activity on the first stages *of C*. *albicans* biofilm formation [25–27].

Third, hLF1-11 inhibited *C*. *albicans* morphogenesis in a dose-dependent manner, in agreement with previous results *in vitro* and *in vivo* [18].

In order to better understand the mechanisms underlying the observed antibiofilm effect of hLF1-11 on *C*. *albicans*, we also investigated the transcriptional profiles of genes associated to cell adhesion, hyphal growth and extracellular matrix production, under biofilm-inducing conditions. The main signaling pathways involved in the induction of hyphae and biofilm formation are the Ras1-cAMP-Efg1 and MAP kinase [5,28,29]. Interestingly, hLF1-11 significantly decreased expression levels of *RAS1*, *CYR1*, *EFG1*, which are members of the Ras1-cAMP-Efg1 pathway. Ras1 GTPase stimulates Cyr1 adenylate cyclase enzyme to synthesize cAMP, which promotes activation of the Efg1 transcription factor. The latter plays an important role in the regulation of some hypha-specific genes, such as *ALS3*, *HWP1* and *ECE1*, which are essential factors of *C*. *albicans* adhesion [30] as well as biofilm formation [29,31–33]. As expected, transcription of *ALS3*, *HWP1* and *ECE1* was also repressed. To confirm these results, exogenous cAMP was added in order to evaluate whether hyphal growth could be restored in hLF1-11-treated *C*. *albicans* cells. The results showed that db-cAMP could partially restore *C*. *albicans* morphogenesis. This finding is in agreement with previous studies showing an antibiofilm activity effect exerted via the inhibition of the Ras1-cAMP-Efg1 pathway [34–36].

In contrast, the hLF1-11 peptide did not affect transcriptional levels of *HST7* and *TEC1* genes. *HST7* codifies a protein kinase belonging to the MAP kinase pathway, whereas *TEC1* encodes a transcription factor that positively regulates the expression of hypha-specific genes, independently from the two pathways described above [37]. On the other hand, *C*. *albicans* morphogenesis is regulated by complex networks responding to a wide variety of extracellular stimuli [38].

Although the composition of the extracellular matrix of *C*. *albicans* biofilm is still not completely characterized, the presence of carbohydrates, proteins and nucleic acids has already been described [39]. The main extracellular carbohydrate identified is β-1,3-glucan, synthesized by the β-1,3-glucan synthase (Gsc1). For this reason, *GSC1* transcript was included as an index of the matrix production [40]. As expected, the hLF1-11 peptide down regulated *GSC1* gene. Yet, little is known about the regulation of biofilm matrix production, which relies upon complex regulatory mechanisms involving several transcription factors. *C*. *albicans* Zap1 (zinc-responsive activator protein) negatively regulated matrix production to promote cellular dispersion. Zap1 directly activates the expression of *CSH1* and *IFD6*, both of which inhibit matrix accumulation, and indirectly represses the expression of other alcohol dehydrogenase genes, such as *ADH5*, which promotes matrix production [41] We found that hLF1-11 reduced transcript levels of *ZAP1* and *CSH1*, whereas it did not influence the expression of *ADH5*. It is worth noting that Zap1 is also required for efficient *C*. *albicans* morphogenesis, in agreement with the finding that *C*. *albicans ZAP1* mutant mainly grows as yeast in biofilm (S1 Fig) [41,42]. Consistently, microscopic examination revealed that hLF1-11-treated *C*. *albicans* cells predominantly grew as yeast after a 24h incubation.

Overall, these results indicate that hLF1-11 inhibits early stages of biofilm formation in *C*. *albicans*, by affecting yeast-hypha transition. In this regard, hLF1-11 seems to be a promising candidate for a potential use as coating agent of prosthetic medical devices, as described for other antimicrobial peptides [43,44].

## Supporting Information

S1 Table Gene-specific primers used for real time qRT-PCR. (PDF)

S1 Fig Summary of peptide induced fluctuation in transcriptional level of biofilm/morphogenesis associated genes. (PDF)
