# Supplementary material for: Inhibition of Candida albicans Biofilm Formation by the Synthetic Lactoferricin Derived Peptide hLF1-11
Source: PLoS One. 2016 Nov 30;11(11):e0167470. doi: 10.1371/journal.pone.0167470 (PMC5130267; doi:10.1371/journal.pone.0167470)
Supplement: S1 Table — (PDF) [file pone.0167470.s001.pdf]

S1 Table

Gene	Forward primer (5'-3')	Reverse primer (5'-3')	Amplified product length (bp)
ACT 1	TAGGTTTGGAAGCTGCTGG	CCTGGGAACATGGTAGTAC	124
ALS 3	GGTTATCGTCCATTTGTTGA	TTCTGTATCCAGTCCATCTT	139
CYR 1	GTTTCCCCCACCACTCA	TTGCGGTAATGACACAACAGA	114
ECE 1	ACAGTTTCCAGGACGCCAT	ATTGTTGCTCGTGTTGCCA	140
EFG 1	TTGAGATGTTGCGGCAGGATA	ACTGGACAGACAGCAGGAC	99
GSC 1	CCCATTCTCTAGGCACGA	ATCAACAACCACTTGCTTCG	117
HST 7	GCCAGTATGGTCGGAGGAT	ACATAGGCATCGTCTTCGTC	67
HWP 1	ACAGGTAGACGGTCAAGG	GGGTAATCATCACATGGTTC	87
RAS 1	GAGGTGGTGGTGTTGGTA	TTCTTCTTGTCCAGCAGTATC	161
TEC 1	GCACTGGCTTCAAGCTCAAA	GCTGCTGCACTCAAGTTCTG	137
ZAP1	ATCTGTCCAGTGTTGTTTGTA	AGGTCTCTTTGAAAGTTGTG	131
ADH5	ACCTGCAAGGGCTCATTCTG	CGGCTCTCAACTTCTCCATA	139
CSH1	CGTGAGGACGAGAGAGAAT	CGAATGGACGACACAAAACA	100
