# Supplementary figures and images for: Inhibition of Candida albicans Biofilm Formation by the Synthetic Lactoferricin Derived Peptide hLF1-11
Source: PLoS One. 2016 Nov 30;11(11):e0167470. doi: 10.1371/journal.pone.0167470 (PMC5130267; doi:10.1371/journal.pone.0167470)

S1 Fig

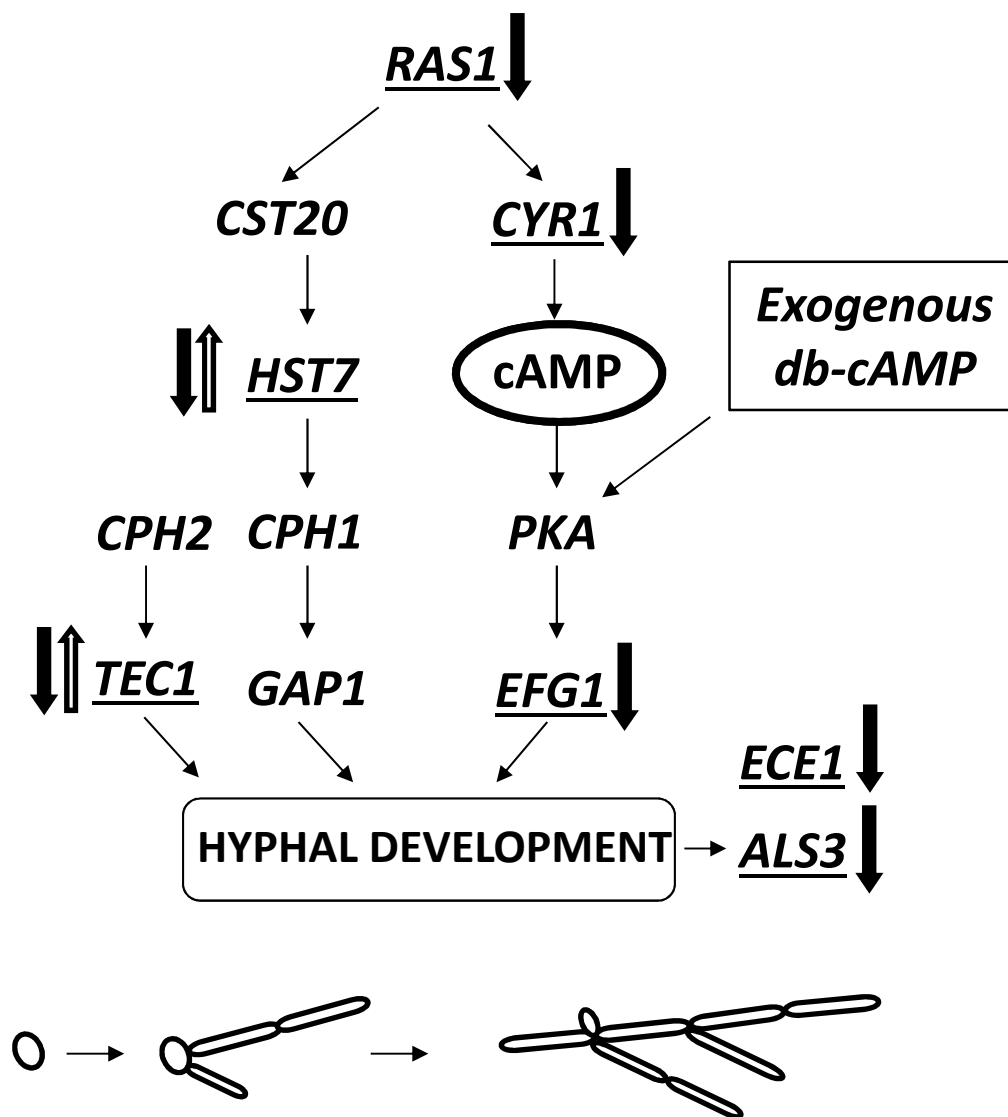

Supplement: S1 Fig — (PDF) [file pone.0167470.s002.pdf]
